# Unmet Nutritional and Psychological Needs of Cancer Patients: An Integrated Multi-Professional Model Approach

**DOI:** 10.3390/diseases10030047

**Published:** 2022-07-21

**Authors:** Giuseppe Carruba, Maria Luisa Calagna, Ildegarda Campisi, Stella Cutrera, Laura Napoli, Giovanni Pitti, Valentina Palmisano, Giuseppina Savio, Antonella Usset, Vita Leonardi, Angela Di Pasquale, Salvatore Requirez, Livio Blasi

**Affiliations:** 1 Servizio di Internazionalizzazione e Ricerca Sanitaria (SIRS), Azienda di Rilievo Nazionale e di Alta Specializzazione (ARNAS)-Civico, Di Cristina, Benfratelli—Palermo, Piazza N. Leotta 2, 90127 Palermo, Italy; 2 Progetto Obiettivo 4.1.5 di Piano Sanitario Nazionale 2015 “Alimentazione e Stili di Vita”, Azienda di Rilievo Nazionale ad Alta Specializzazione (ARNAS)-Civico, Di Cristina, Benfratelli, Piazza N. Leotta 2, 90127 Palermo, Italy; luisa.calagna@gmail.com (M.L.C.); ildecampisi@gmail.com (I.C.); stella.cutrera@gmail.com (S.C.); info@nutrizionistalauranapoli.it (L.N.); giannipitti@gmail.com (G.P.); valentina.palmisano@arnascivico.it (V.P.); 3 Clinical Oncology, Medical Oncology Unit (ARNAS)-Civico, 90100 Palermo, Italy; giuseppe.savio@arnascivico.it (G.S.); antonella.usset@arnascivico.it (A.U.); vitabaldassara.leonardi@arnascivico.it (V.L.); livio.blasi@arnascivico.it (L.B.); 4 Azienda di Rilievo Nazionale ad Alta Specializzazione (ARNAS)-Civico, Di Cristina, Benfratelli, Piazza N. Leotta 2, 90127 Palermo, Italy; angela.dipasquale@arnascivico.it; 5 Health Department, Azienda di Rilievo Nazionale ad Alta Specializzazione (ARNAS)-Civico, Di Cristina, Benfratelli, Piazza N. Leotta 2, 90127 Palermo, Italy; direzione.sanitaria@arnascivico.it

**Keywords:** cancer patients, nutritional status, psychological status, multi-professional interventions, model approach

## Abstract

**Simple Summary:**

This paper reports the design and exploitation of an integrated approach aimed at addressing the nutritional and psychological needs of cancer patients. In particular, patients recruited for the study undertook a path through which their nutritional and psychological status was assessed, improved and reinforced, ultimately leading to ameliorating their quality of life and, eventually, the outcome of their disease.

**Abstract:**

This paper presents a multi-professional integrated approach toward the recognition and management of the nutritional and psychological needs of cancer patients. In particular, the patients undertook a multi-professional, multistep process that included the collection of both personal and clinical data, the evaluation of anthropometric measures, nutritional status and psychometric indices, and an ensuing personalized nutritional prescription and psychological support, ultimately leading to combined nutritional and psychological interventions to control their adherence to a nutritional program and to consolidate motivation to change. Overall, 120 patients were recruited for the study. The majority (84.2%) were female. Breast cancer was by far the most frequent malignancy (52.5%), followed by colorectal (17.5%), pancreatic (9.2%), ovarian (9.2%) and lung (5.0%) cancers. The results of the nutritional and psychological screening at baseline indicated that only 35% of patients had a normal BMI, whilst a relatively high proportion (nearly 32%) was overweight or obese (25%). The INRAN and MEDI-LITE questionnaires, which were used to assess the eating habits and adherence to a Mediterranean diet, respectively, revealed a mixed prevalence of cereals/cereal-based, fresh/processed meat, and fish or fishery food, with a medium–low adherence to the Mediterranean diet in nearly 38% of patients. The BUT, HADS and SF-36 tests, which were used to assess psychological disturbances, showed that 37.5% of patients had disorders regarding body image, 29.2% had abnormal anxiety and 20.0% had a depressive state, while no significant association was observed between the SF-36 PCS and MCS and the patients′ characteristics. The results of the potential impact of this novel approach on the QoL of patients after completion of the course are awaited with expectation.

## 1. Introduction

Cancer is a foremost cause of death and a major factor in reducing life expectancy worldwide. There is evidence that cancer may surpass cardiovascular diseases as the leading cause of premature death in the vast majority of countries within the present century [1,2]. According to the GLOBOCAN, which is produced by the International Agency for Research on Cancer (IARC), global rates of cancer incidence and mortality were found to increase when comparing 2018 data with 2020 data, respectively rising from 18.1 to 19.3 million new cases and from 9.6 to nearly 10.0 million deaths [3,4]. This epidemiologic behavior implies that cancer has a continual and ever-growing harmful effect not only in terms of the health status of the population but also on even larger psychological, relational and socio-economic societal aspects.

Cancer is a complex, multi-factorial disease that has a profound impact on the nutritional status and psychological/relational dimensions of patients and their caregivers. In this framework, considerable literature supports the concept that lifestyle and, notably, diet play an important role in determining both the potential risk of developing cancer in healthy individuals and the clinical outcome of the disease in cancer patients [5,6,7]. In this context, the World Cancer Research Fund (WCRF), jointly with the American Institute for Cancer Research (AICR), recently published the Third Expert Report on “Diet, Nutrition, Physical Activity and Cancer: a Global Perspective”, which provides an extensive and updated review of current evidence relating diet, nutrition and physical activity to cancer, as well as an expert review of potential mechanisms underlying the causal link between exposure and risk of developing cancer [8]. In a recent systematic review and meta-analysis, Solans et al. evaluated the 2007 WCRF/AICR score in relation to cancer-related health outcomes, concluding that adherence to the 2007 WCRF/AICR recommendations results in a reduced risk of cancer incidence and mortality [9].

The nutritional status of cancer patients is profoundly altered by either the disease itself or the adjuvant/advanced chemotherapy regimens. Both the nature and extent of this alteration are strictly dependent on the tumor type and localization and its stage, along with a large variety of factors related to individual conditions, both clinical and psychological. Modifications of the nutritional status of cancer patients often include sarcopenia, malnutrition and cachexia, whose definition, underlying mechanisms and differential diagnosis were the focus of a recently published review [10]. On the other hand, anticancer therapies, such as anti-metabolites and platinum-based drugs, are responsible for both nutritional deficiencies and inappropriate dietary intakes in a considerable proportion of cancer patients [11]. In addition, chemotherapy frequently induces an array of side effects, including inappetence, modifications of taste and smell, mouth inflammation and dryness, nausea, vomiting, diarrhea and asthenia, that may significantly contribute to producing a poor nutritional intake in cancer patients [12,13,14].

From another standpoint, cancer heavily impacts psycho-social and relational aspects of individual patients, while anxiety and depression may in turn negatively affect both their nutritional status and quality of life. In particular, cancer produces drastic changes in most aspects of patients’ life, including familial, work, social and financial environments, with a differential prevalence of problems and emotional status across the diagnosis, chronic and terminal stages of the disease [15]. Furthermore, cancer patients are often confronted with a large assortment of needs that may be disparate and depend on the individual condition, history, personality and perception of the disease. According to the Italian Association of Medical Oncology, nearly 20% of cancer patients suffer from depression, 10% from anxiety and up to 50–60% are burdened by psychological distress [16]. Furthermore, cancer patients commonly experience psychosocial problems, including post-traumatic stress, fatigue, sexual dysfunction and cognitive impairment [17]. In an individual patient data meta-analysis, Kalter et al. [18] reported that psychosocial interventions (PSI) significantly improved the quality of life (QoL) and emotional and social function of cancer patients, with the distinct effects depending on a variety of personal, clinical, demographic and intervention-specific characteristics. This suggests that PSI on patients with cancer should be individually tailored and integrated into a comprehensive approach that includes, along with conventional anti-cancer therapies, nutritional, psychological and emotional support, appropriate medical information, and communication for both patients and caregivers. In fact, in a holistic approach, every patient should be considered as a whole with their familial, social and work environment, with the aim of shaping interventions directed at promoting wellbeing, improving quality of life, relieving symptoms of disease and restricting side-effects of conventional treatments in individual patients. In this framework, psychological support represents an essential component of integrated, targeted approaches aimed at ameliorating the quality of life of cancer patients. Notwithstanding, today, both nutritional and psychological support are quite often neglected or even ignored in most clinical oncology units of major cancer hospitals globally.

In this paper, we describe the modeling and implementation of a multi-professional, integrated approach based on the detection of discomfort and the nutritional and psychological needs of cancer patients through validated and standardized instruments and the ensuing management and preservation of dietary and lifestyle changes using innovative tools, such as multidisciplinary support groups, ultimately leading to developing a structured methodology approach toward curing the patient and its centrality in the healthcare processes.

## 2. Patients and Methods

All patients recruited in this project attended the Clinical Oncology Unit at the ARNAS-Civico healthcare center from September 2020 up to July 2021. The one and only exclusion criterion concerned patients in the terminal phase of their disease, as recently defined [19].

**Figure 1:**
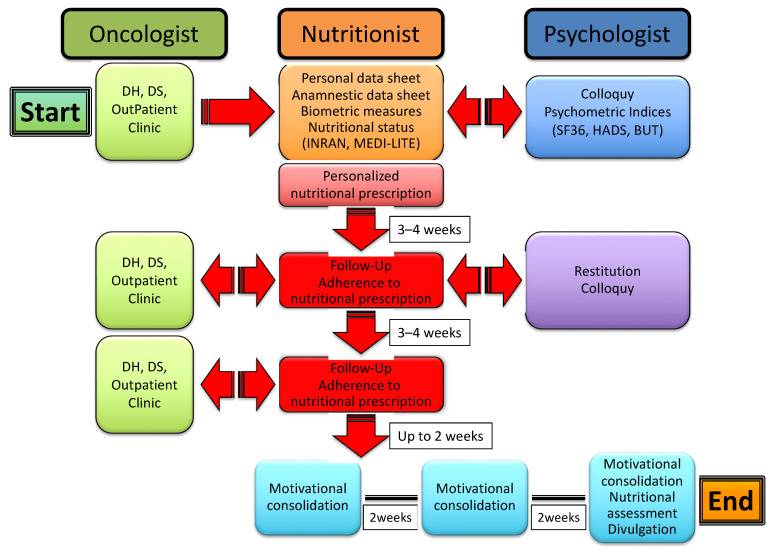
The multi-professional, multistep process of nutritional and psychological support for cancer patients.

Patients enrolled in the study undertook a multi-professional, multistep process that included the collection of both personal and clinical data, the evaluation of anthropometric measures, nutritional status and psychometric indices, and an ensuing personalized nutritional prescription along with psychological support, eventually leading to follow-up of the patients to control their adherence to the nutritional program and to consolidate motivation to change through support groups. The whole process is illustrated in Figure 1.

In particular, the patients were led by an oncologist to a nutritionist and thereafter recruited in a preliminary meeting whereby they were interviewed, received an informative note, and signed the informed consent for acceptance and adhesion to the study. Personal and anamnestic data were collected through a specifically designed data sheet; socio-demographic information, lifestyle and eating habits were evaluated through an INRAN (Istituto Nazionale di Ricerca per gli Alimenti e la Nutrizione) questionnaire that was developed based on the Italian National Food Consumption Survey INRAN-SCAI 2005-06 [20]; and the adherence to a Mediterranean dietary model was assessed through a modification of the MEDI-LITE scoring system [21]. Anthropometric indices (body mass index (BMI) and waist-to-hip ratio (WHR)) were calculated through measurements of weight, height, waistline and hipline. Potential pharmacological treatment for concurrent chronic diseases, including hypertension, hypercholesterolemia, hypertriglyceridemia and diabetes, was also recorded. Patients were then sent to a psychologist for a preliminary colloquy and the subsequent administration of psychometric tests, including SF36 [22], HADS [23] and BUT [24].

**Figure 2:**
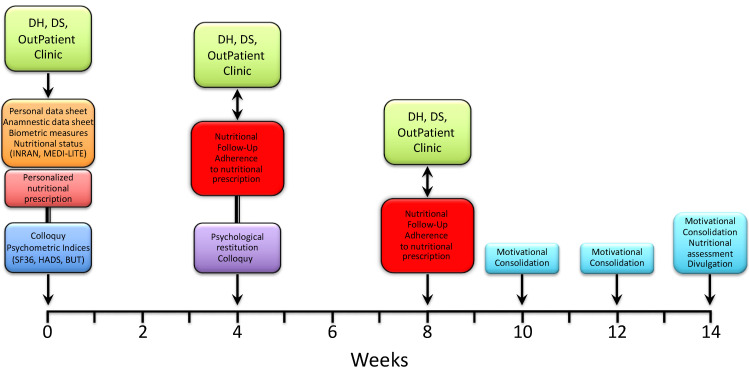
Timeline of the nutritional and psychological intervention path.

Immediately after, the patients were met again by a nutritionist to receive and be instructed on a personalized nutritional prescription. The latter was commonly divided into 5 meals (breakfast, snack, lunch, snack, dinner), with each comprising an assortment of options and specific indications based on both individual health status and nutritional principles of the Mediterranean diet. After 3–4 weeks, patients were reevaluated by a nutritionist for both the measurement of anthropometric indices and the adherence to nutritional prescription. The latter was assessed through a scoring system whereby each of the 5 meals were scored 0 to 2 depending on null/limited, intermediate and high adherence, respectively; the total score was expressed as a percent of the maximum achievable score (i.e., 10). Patients were then sent to a psychologist for a collection of psychometric tests and a further colloquy aimed at the constraint of potential resistances, either individual or familial, and to the release of psycho-physical resources with the purpose of facilitating the adaptation to a nutritional program and promoting lifestyle changes. After 3–4 weeks, the patients underwent a second follow-up meeting with a nutritionist to assess the anthropometric measures and adherence to the nutritional prescription. After a variable time interval (up to 2 weeks), patients were included in three small, multi-professional intervention groups, also comprising a nutritionist and a psychologist, which were aimed at further consolidating the lifestyle and dietary changes through the confrontation of difficulties and/or resistances experienced and capabilities or resources developed by patients along the treatment pathway. The three groups were scheduled every 2 weeks, with the final one including both nutritional (INRAN, MEDI-LITE, adherence to prescription) and psychological (SF-36, HADS, BUT) re-appraisal of the patients. As illustrated in Figure 2, the whole treatment pathway ran for a period of 11 to 14 weeks.

At this stage of the study, statistical analysis was limited to the 120 subjects recruited in the period June 2020–July 2021. 

*Statistics*. Data related to patient characteristics and study variables were expressed using frequency (%) and mean ± standard deviation or median and interquartile ranges unless otherwise stated. In order to determine the association between the results of the food questionnaires and psychometric tests and the general characteristics of patients at the baseline, the statistical analysis was conducted using the chi-squared test (including the Cramer′s V test) and the permutation test by gender and residence by simulating 100,000 samples extracted with the Monte Carlo method for the approximation of the exact conditional distribution.

In addition, the Kruskal–Wallis test (one-way non-parametric ANOVA) was used for the analysis of age groups, educational level, BMI classes and types of cancer. Finally, the Kendall rank correlation test was used to evaluate the correlations between PCS and MCS and the other variables under study.

The statistical analysis was performed using the STATA software v.13 (StataCorp LLC, College Station, TX, USA) and an alpha level of 0.05 was used for all tests.

## 3. Results

Overall, 120 patients were recruited for the study, all of which were attending the Clinical Oncology Unit at the Azienda di Rilievo Nazionale e di Alta Specializzazione-Civico, Di Cristina, Benfratelli (ARNAS-Civico), from September 2020 to July 2021. The greater part (n = 101, 84.2%) of patients were female, while a minority (n = 19, 15.8%) of male subjects entered the study. The large preponderance of female subjects was mostly the consequence of the historically high number of breast cancer cases attending the ARNAS-Civico and of the large Breast Unit working in the context of the Oncology Department.

The average age ranged from 27 to 74 years for female subjects and from 40 to 77 years for male subjects, with an overall median age of 56 ± 10.5 years.

As reported in Table 1, the study subjects were also subdivided according to their place of residence, degree of education, anthropometric measures (BMI, WHR) and tumor type.

Almost the totality of patients resided in cities (95.8%) and the majority (65%) fell in the secondary class of education, including secondary and high school.

It is noteworthy that only 35% of patients presented a normal BMI, whilst a relatively high proportion (nearly 32%) was overweight or even fell into the obesity ranges (25% as a whole), with a significant 8.3% scoring over 40 in the obesity class III. Measurement of the waist-to-hip ratio (WHR) as an indicator of obesity and the ensuing risk of developing chronic diseases, including cancer, confirmed the rather large prevalence of obese subjects, with proportions of 67.3% and 63.2% obese subjects among the females and males, respectively.

As indicated in the Patients and Methods section, the eating habits of the study subjects were assessed using the INRAN questionnaire, whereby the frequency of consumption of 15 selected food groups was divided into *never*, *yearly*, *monthly*, *weekly* and *daily* categories. In particular, the following groups were classified: cereals and derivatives, cereal-based products, fresh meat, processed meat, fish or fishery products, milk and/or yogurt, fresh fruit, dried fruit, vegetables or greens, legumes, eggs, sweets, carbonated water/sweetened drinks and alcoholic beverages.

As illustrated in Table 2, a large proportion of patients reported daily consumption of cereals and derivatives (over 98%) and, though to a lesser extent, of cereal-based products (>78%); on the other hand, weekly consumption of fresh and processed meat was reported by the majority of subjects (>90% and 55%, respectively). A weekly intake of fishery food was reported in a considerable percentage (84.2%) of patients. The consumption of milk/yogurt and dairy products was very common, attaining a daily 57.5% and a weekly 61.7%, respectively. The daily consumption of fresh fruit and vegetables/greens was reported in high percentages of study subjects at 84.1% and 65.8%, respectively. Equally large proportions of subjects reported a weekly intake of legumes (74.2%) and eggs (81.7%). A variable consumption of both dried fruit and sweets was reported across all study subjects. No or uncommon intake of carbonated/sweetened drinks and alcoholic beverages was reported by the majority of patients.

The MEDI-LITE score was originally developed through the analysis of data generated by numerous cohort studies that investigated the association between adherence to the Mediterranean diet and health outcomes [25]. In this system, nine food categories are considered. In the case of food groups typical of the Mediterranean diet (fruit, vegetables, cereals, legumes, fish and olive oil), the highest category of consumption scores 2, while the middle and the lowest categories respectively score 1 and 0. Conversely, for the food groups not typical of the Mediterranean diet (meat and meat products, milk and dairy products), a value of 2 is assigned to the lowest category, 1 to the middle and 0 to the highest. Based on the final score (range of 0–18), obtained as the sum of values relevant to any individual food group, study subjects were divided into two classes: medium–low adherence (scoring 0 to 9) and medium–high adherence (scoring 10 to 18).

Out of the 120 patients studied, n = 75 (62.5%) fell in the medium–high adherence category, whilst n = 45 (37.5%) showed a medium–low adherence to the Mediterranean diet. There was no significant association between the MEDI-LITE score and other individual characteristics, including age, degree of education and occupation, except for a slight prevalence of medium–high adherence score in employees as compared with housewives (data not shown).

Psychometric tests used in this study included the Body Uneasiness Test (BUT) [24], the Hospital Anxiety and Depression Scale (HADS) [23] and the Short Form Health Survey 36 (SF-36) [22], as described elsewhere. The results are reported in Table 3. Furthermore, the statistical analysis of the potential association between psychometric indices and patients’ characteristics is reported in Table 4.

In particular, the BUT test was used to assess body image disorders in the study subjects, with the latter being subdivided into two categories, *minor* and *noteworthy*, depending on the gravity of the condition. Overall, a significant proportion (37.5%) of patients presented a pathological perception of body image, with a greater prevalence in female (40.6%) than in male (21.1%) subjects (see Table 3). As expected, an increasingly high percentage of body image disorders was observed in patients with respect to BMI, rising from normal weight (34.9%) to overweight (40.5%) and obese (46.7%) subjects. A slightly higher prevalence of body image disorders was revealed in patients living in urban areas (40.9%) relative to those residents in villages in the countryside (28.1%) and in subjects with secondary or higher education degrees (44.6% as a whole) relative to those having primary education only (29.1%). Predictably, an alteration in body image perception was largely prevalent (28/60, 46.7%) in patients with breast cancer as compared with other tumor types (17/60, 28.3%).

The HADS test, which is based on a self-assessment scale, was found to provide a reliable instrument for identifying and measuring depression and anxiety states in a hospital outpatient setting. In particular, based on the presence and severity of the emotional disorder, each subject was classified as *normal*, *borderline* or *abnormal*. In our study, a considerable proportion of patients presented an abnormal anxiety level (29.2%) and, though to a lesser extent, a depressive (20.0%) state (see Table 3).

Interestingly enough, the HADS anxiety state was significantly associated with age (*p* = 0.003, Pearson χ^2^ and Cramer′s V tests). Furthermore, the presence of a remarkably greater percentage of abnormal anxiety states was observed in patients living in cities (33%) as compared with patients residing in villages (18.7%), as well as in subjects having a primary (34.5%) or secondary (31.7%) degree of education relative to subjects with a higher education (12.5%). No significant difference was revealed when comparing different BMI and tumor types, except for a trend toward an increasingly high abnormal anxiety state from underweight (20.0%) to overweight (32.4%) patients.

As far as the HADS depression scale was concerned, a highly significant greater percentage of abnormal depression was observed in male (36.8%) relative to female (16.8%) subjects (*p* < 0.001). Once again, patients living in villages had a lower incidence of abnormal depression relative to those living in cities (9.4% vs. 23.9%, respectively), as well as in patients with a higher education level (16.7%) compared with patients with primary (21.8%) or secondary (19.5%) education degree. No remarkable difference was observed when comparing different BMIs, while breast cancer patients exhibited a lower percentage of abnormal depression (15.0%) relative to patients with other tumor types (25.0% as a whole).

The Short Form Health Survey 36 (SF-36) was used as a versatile tool for measuring health-related quality of life (HRQOL) in study subjects. The SF-36 comprises eight domains, precisely: physical functioning (PF), role physical limitations (RP), bodily pain (BP), general health (GH), vitality (VT), social functioning (SF), role emotional limitations (RE) and mental health (MH). In addition, two summary components, namely, physical (PCS) and mental (MCS), are also included respectively to combine the PF, RP, BP and GH domains on one hand and to encompass the VT, SF, RE and MH domains on the other. Understandably, the two components have some degree of overlap, with the VT, GH and SF domains having significant implications in both components. The results of the SF-36 PCS and MCS are illustrated in Table 3. Overall, no significant association was observed between the PCS and MCS and the patients′ characteristics considered, although a trend was observed for the PCS with tumor type (*p* = 0.098) and for the MCS with gender (*p* = 0.078).

## 4. Discussion

Epidemiological studies clearly indicate that the epidemics of NCDs that Western countries have witnessed in the last few decades represent the result of the dramatic changes that have occurred in both food systems and consumer eating habits and behavior globally [26,27,28]. This critical issue is also related to the actual sustainability of both the consumption and production of healthy food [29]. In particular, the European Prospective Investigation into Cancer and Nutrition (EPIC) program has highlighted the strong association between lifestyle, food environment and nutritional behavior with cancer incidence [30]. In addition, nutrition plays a key role not only in cancer prevention but also in disease progression and recurrence, as well as in treatment tolerance.

Notwithstanding, as emphasized by Rauh et al. [31], nutritional issues are very often neglected and/or poorly structured in the clinical care of cancer patients. In this context, the authors underlined the concept that nutritional assessment and management should become an integrative part of clinical trials in oncology, involving the use of nutrition specialists in an interdisciplinary approach and eventually leading to interventions that are diversified according to individual needs.

In the present study, the proposed multi-professional approach was modeled with the aim to obtain an initial recognition of the nutritional status and behavior of individual patients and to tailor the nutritional prescription to the specific necessities of the study subjects and their environment. Furthermore, both the adherence to the prescription, as assessed through a simple scoring system, and its efficacy, also determined through the measurement of anthropometric indices, were revaluated monthly using a nutritional follow-up (for details see the Patients and Methods section). This allowed not only to verify whether the nutritional prescription was being correctly complied with by the patients but also to introduce changes that may eventually become desirable. 

Nutritional issues represent an important aspect of clinical oncology, with a considerable proportion of cancer patients having nutritional impairment [32]. The latter is a complex condition, ranging from overweight/obesity to an increasingly high percentage of weight loss and sarcopenia and/or cachexia during the clinical course of the disease. In our study, a relatively large proportion of both female and male patients were obese (25%) or overweight (32%) at presentation. This evidence was corroborated by the finding that a considerable percentage of patients (nearly 38%) revealed a medium–low adherence to the Mediterranean diet, as assessed through the MEDI-LITE scoring system.

Doubtlessly, nutrition has a profound impact on quality of life (QoL) from both physical and psychological standpoints. From a holistic perspective, clinical care of cancer patients should integrate appropriate scrutiny and customized interventions regarding individual nutritional, psychosocial and relational issues. In an important position paper, the European Society for Medical Oncology (ESMO) recently introduced the term “patient-centered care” to identify an integrated approach of both supportive and palliative care implemented along the disease course, whereby a multidisciplinary approach is systematically exploited to meet the composite and variable patients’ needs [33]. In this context, our multi-professional model allows an interoperable and continuous flow of information between an oncologist, nutritionist and psychologist through which clinical data is used to adapt nutritional assessment and counseling, individual nutritional issues are incorporated to shape psychological appraisal of individual patients, and both nutritional and psychological profiles are combined in intervention groups and interpolated with clinical data to determine their ultimate impact not only, broadly, on QoL but also the outcome of the disease. In the present study, data obtained at baseline supported the concept that cancer patients are deeply affected by a variety of psychosocial problems, including disorders of body image (37.5%), anxiety (29.2%) and, though to a lesser extent, depression (20.0%), with a further significant proportion being classified as borderline for anxiety (44.2%) and depression (38.3%). Furthermore, we observed that the psychological disturbances we considered, namely, body image disorders, anxiety and depression, were related to intrinsic patient characteristics, including gender, BMI, place of residence and education level. This implied that the combination of clinical, nutritional and psychological interventions on cancer patients should also include the evaluation of the above individual and environmental features to ultimately lead to a personalized, integrated approach to cancer care.

Nevertheless, quite frequently, if not regularly, all psychological and psychosocial disturbances are neither considered nor assisted in the clinical practice. Recently, in a population survey, Lewandoska et al. [15] reported that a relatively high proportion (46%) of cancer patients revealed a variable degree of despair, depression and feelings of helplessness, and that 93% of patients felt being in need of help regarding various aspects.

Furthermore, there is convincing evidence that the levels of anxiety and depression of cancer patients are strictly interconnected with their nutritional status, depicting a sort of vicious cycle by which psychological distress may result in disturbances of nutritional behavior and vice versa [34]. In our study, the integration of nutritional scrutiny, intervention and follow-up, combined with tackling the psychosocial needs of cancer patients, is expected to create a positive, patient-centered environment whereby individual patients would become proactive protagonists of their disease, ultimately leading to improved quality of life and to ease the complex interactions between healthcare providers, patients and caregivers.

Another issue that has become extremely relevant during the last two decades is the overload of the information environment on cancer and nutrition [35]. This may eventually lead to severely hampering a correct understanding and appropriate knowledge of evidence-based principles in the field of both cancer prevention and care. We have accorded special attention to this sensitive problem not only by accurately selecting the sources of information used for nutritional counseling but also through the design and production of informative material on nutrition, cancer prevention and cancer treatment that we have distributed to cancer patients and circulated among their caregivers with the aim to create an information environment that could assist in developing an adequate level of acquaintance and awareness in patients and close relatives or assistants.

## 5. Conclusions

In conclusion, despite this study being burdened by some limitations (small size, heterogenous population), it however proposed an innovative model that was aimed at capitalizing on the integration of clinical, nutritional and psychological interventions into a united, holistic approach to cancer care.

The data obtained at baseline clearly indicated that, in a significant proportion of cases, the cancer patients presented with a large variety of physical, nutritional and psychosocial problems that required an individually tailored, integrated intervention and regular follow-ups to secure and consolidate the changes accomplished.

This integrated model may become a practice that could be replicable and implemented throughout the majority of clinical settings in oncology centers, eventually leading not only to improve the quality of life of cancer patients but also to having a positive effect on both their familial and psychosocial environments and the clinical course of the disease.

The results of the potential impact of this novel approach on both the QoL and clinical outcome after the course was completed for all patients are awaited with expectation.

## Figures and Tables

**Table 1:** Patients recruited for the study: descriptive statistics.

Age	25–45	46–65	66–85				
	22 (18.3%)	77 (64.2%)	21 (17.5%)				
							
**Sex**	F	M					
	101 (84.2%)	19 (15.8%)					
							
**Place of residence**	City	Village					
	115 (95.8%)	5 (4.2%)					
							
**Education**	Primary	Secondary	Higher				
	18 (15.0%)	78 (65.0%)	24 (20.0%)				
							
**BMI**	Underweight	Normal	Overweight	Obese I	Obese II	Obese III	
	10 (8.3%)	42 (35.0%)	38 (31.7%)	15 (12.5%)	5 (4.2%)	10 (8.3%)	
							
**WHR**	Female			Male			
	Normal	Over	Obese	Normal	Over	Obese	
	12 (11.9%)	21 (20.8%)	68 (67.3%)	3 (15.8%)	4 (21.0%)	12 (63.2%)	
							
**Tumor type**	BC	LC	CRC	PC	OC	GC	Other
	63 (52.5%)	6 (5.0%)	21 (17.5%)	11 (9.2%)	11 (9.2%)	3 (2.5%)	5 (4.1%)

Values represent the number and percent distributions of the cancer patients’ characteristics.

**Table 2:** The INRAN questionnaire: frequencies of consumption for different food groups.

		Frequency				
	Food Group	Never	Yearly	Monthly	Weekly	Daily
1.	Cereals and derivatives (pasta, rice, bread, pizza, spelt, barley, etc.)	0	0	0	2 (1.7)	118 (98.3)
2.	Cereal-based products (cornflakes, biscuits, rusks, crackers, etc.)	11 (9.2)	1 (0.8)	3 (2.5)	11 (9.2)	94 (78.3)
3.	Fresh meat	3 (2.5)	1 (0.8)	7 (5.8)	109 (90.9)	0
4.	Processed meat (ham, salami, wurstels, etc.)	21 (17.5)	2 (1.7)	27 (22.5)	66 (55.0)	4 (3.3)
5.	Fish or fishery products (sea bass, octopus, shrimps, etc.)	3 (2.5)	0	16 (13.3)	101 (84.2)	0
6.	Milk and/or yogurt	33 (27.5)	1 (0.8)	3 (2.5)	14 (11.7)	69 (57.5)
7.	Dairy products (e.g., fresh or aged cheese)	8 (6.7)	0	7 (5.8)	74 (61.7)	31 (25.8)
8.	Fresh fruit	3 (2.5)	0	2 (1.7)	14 (11.7)	101 (84.1)
9.	Dried fruit (walnuts, almonds, hazelnuts, etc.)	33 (27.5)	4 (3.3)	19 (15.8)	26 (21.7)	38 (31.7)
10.	Vegetables or greens (lettuce, chicory, endive, spinach, chard, etc.)	8 (6.7)	0	3 (2.5)	30 (25.0)	79 (65.8)
11.	Legumes (beans, lentils, peas, chickpeas, etc.)	11 (9.2)	1 (0.8)	17 (14.1)	89 (74.2)	2 (1.7)
12.	Eggs	8 (6.7)	1 (0.8)	9 (7.5)	98 (81.7)	4 (3.3)
13.	Sweets (cakes, ice-cream, chocolate)	21 (17.5)	3 (2.5)	20 (16.7)	43 (35.8)	33 (27.5)
14.	Carbonated and/or sweetened drinks	72 (60.0)	2 (1.7)	11 (9.2)	20 (16.6)	15 (12.5)
15.	Alcoholic beverages (wine, beer, spirits, etc.)	82 (68.4)	1 (0.8)	10 (8.3)	15 (12.5)	12 (10.0)

Values represent the number and percent distributions of consumption frequencies for the different food groups considered.

**Table 3:** Distribution and associations of the BUT, HADS and SF-36 components with patient characteristics.

		BUT *n* (%)		HADS Anxiety *n* (%)			HADS Depression *n* (%)			SF-36 *Mean* (±SD)	
		Minor	Noteworthy	Normal	Borderline	Abnormal	Normal	Borderline	Abnormal	PCS	MCS
**Age**	25–45	14 (60.9)	9 (39.1)	7 (30.4)	9 (39.2)	7 (30.4)	9 (39.1)	9 (39.1)	5 (21.8)	36.0 (8.6)	40.3 (11.9)
	46–65	46 (60.5)	30 (39.5)	21 (27.6)	33 (43.4)	22 (29.0)	35 (46.1)	28 (36.8)	13 (17.1)	38.5 (9.5)	39.2 (9.5)
	66–85	15 (71.4)	6 (28.6)	4 (19.10)	11 (52.4)	6 (28.6)	6 (28.6)	9 (42.8)	6 (28.6)	37.7 (6.3)	36.8 (10.3)
											
**Sex**	F	60 (59.4)	41 (40.6)	25 (24.8)	47 (46.5)	29 (28.7)	44 (43.6)	40 (39.6)	17 (16.8)	39.3 (9.0)	38.9 (10.3)
	M	15 (78.9)	4 (21.1)	7 (36.8)	6 (31.6)	6 (31.6)	6 (31.6)	6 (31.6)	7 (36.8)	36.4 (7.7)	39.2 (9.5)
											
**Place of residence**	City	52 (59.1)	36 (40.9)	20 (22.7)	39 (44.3)	29 (33.0)	34 (38.6)	33 (37.5)	21 (23.9)	37.4 (8.8)	38.8 (10.0)
	Village	23 (71.9)	9 (28.1)	12 (37.5)	14 (43.8)	6 (18.7)	16 (50.0)	13 (40.6)	3 (9.4)	39.3 (9.0)	39.5 (10.7)
											
**Education**	Primary	39 (70.9)	16 (29.1)	14 (25.5)	22 (40.0)	19 (34.5)	20 (36.4)	23 (41.8)	12 (21.8)	36.9 (8.6)	38.5 (8.6)
	Secondary	22 (53.6)	19 (46.3)	10 (24.4)	18 (43.9)	13 (31.7)	21 (51.2)	12 (29.3)	8 (19.5)	38.4 (10.1)	39.7 (11.9)
	Higher	14 (58.3)	10 (41.7)	8 (33.3)	13 (54.2)	3 (12.5)	9 (37.5)	11 (45.8)	4 (16.7)	39.5 (7.0)	38.8 (10.4)
											
**BMI**	Underweight	9 (90.0)	1 (10.0)	3 (30.0)	5 (50.0)	2 (20.0)	4 (40.0)	6 (60.0)	0 (0.0)	33.0 (5.6)	37.9 (8.0)
	Normal	28 (65.1)	15 (34.9)	10 (23.3)	20 (46.5)	13 (30.2)	21 (48.9)	13 (30.2)	9 (20.9)	39.0 (9.4)	39.3 (10.0)
	Overweight	22 (59.5)	15 (40.5)	10 (27.0)	15 (40.5)	12 (32.4)	12 (32.4)	18 (48.7)	7 (18.9)	37.5 (9.1)	37.6 (11.1)
	Obese I	7 (46.7)	8 (53.3)	3 (20.0)	5 (33.3)	7 (46.7)	2 (13.3)	6 (40.0)	7 (46.7)	40.1 (9.7)	40.9 (12.2)
	Obese II	4 (80.0)	1 (20.0)	3 (60.0)	2 (40.0)	0 (0.0)	5 (100.0)	0 (0.0)	0 (0.0)	39.1 (6.4)	41.4 (7.5)
	Obese III	5 (50.0)	5 (50.0)	3 (30.0)	6 (60.0)	1 (10.0)	6 (60.0)	3 (30.0)	1 (10.0)	35.5 (6.2)	39.4 (7.5)
											
**Tumor type**	BC	32 (53.3)	28 (46.7)	15 (25.0)	28 (46.7)	17 (28.3)	29 (48.3)	22 (36.7)	9 (15.0)	39.0 (9.4)	39.8 (10.3)
	LC	5 (83.3)	1 (16.7)	1 (16.7)	4 (66.6)	1 (16.7)	3 (50.0)	2 (33.3)	1 (16.7)	36.9 (6.8)	33.3 (7.0)
	CRC	15 (75.0)	5 (25.0)	9 (45.0)	5 (25.0)	6 (30.0)	9 (45.0)	7 (35.0)	4 (20.0)	38.7 (9.7)	42.7 (10.2)
	PC	7 (63.6)	4 (36.4)	2 (18.2)	4 (36.4)	5 (45.4)	1 (9.1)	6 (54.5)	4 (36.4)	35.1 (5.8)	36.1 (9.7)
	OC	7 (70.0)	3 (30.0)	4 (40.0)	4 (40.0)	2 (20.0)	4 (40.0)	3 (30.0)	3 (30.0)	36.0 (9.3)	36.9 (9.4)
	GC	4 (100.0)	0 (0.0)	1 (25.0)	1 (25.0)	2 (50.0)	2 (50.0)	1 (25.0)	1 (25.0)	40.1 (7.5)	31.7 (12.6)
	Other	5 (55.6)	4 (44.4)	0 (0.0)	7 (77.8)	2 (22.2)	2 (22.2)	5 (55.6)	2 (22.2)	33.9 (6.6)	37.5 (8.8)

Data represent the number and percent distributions of the BUT and HADS parameters and the means ± standard deviations (SD) of SF-36 physical and mental component summaries of the 120 cancer patients recruited for the study.

**Table 4:** Statistical analysis of the association between the psychometric indices and patients’ characteristics.

		HADS		SF-36	
	BUT	Anxiety	Depression	PCS	MCS
**Age**	0.106	0.421	<0.001	0.187	*0.078*
**Sex**	0.648	0.003	0.820	0.367	0.345
**Place of residence**	0.201	0.170	0.420	0.654	0.324
**Education**	0.199	0.381	0.206	0.435	0.432
**BMI**	0.267	0.574	*0.092*	0.654	0.877
**Tumor type**	0.283	0.313	0.221	*0.098*	0.987

Data represent *p*-values calculated using the chi-squared test (with the Cramer′s V test: for details see the Patients and Methods section). Values in italics indicate a trend; values in bold identify those that were statistically significant (*p* < 0.05).

## Data Availability

The authors confirm that, according to the FAIR principles (findability, accessibility, interoperability, reproducibility) for data access, the data supporting the findings of this study are available within the article. Furthermore, all supporting data and preprocessing/analysis code, as well as any previously unreported software/bioinformatic tool, will be made available to editors and peer reviewers upon request to the corresponding author for the purposes of evaluating the manuscript.
